# Unmasking Juvenile Systemic Lupus Erythematosus (JSLE): Multisystemic Manifestations in an Adolescent Male, A Rare Case Report

**DOI:** 10.1002/ccr3.72465

**Published:** 2026-04-07

**Authors:** Shafaq Nazia, Aliza Andaleeb, Abdul Moiz, Hareem Ahmed, Muhammad Hassaan Javaid, Abubakr Mahmoud

**Affiliations:** ^1^ Department of Medicine Liaquat University of Medical Health Sciences Jamshoro Pakistan; ^2^ Department of Medicine Bond University Queensland Australia; ^3^ Department of Medicine Shifa College of Medicine Islamabad Pakistan; ^4^ University of Khartoum Faculty of Medicine Khartoum Sudan

**Keywords:** autoimmune, juvenile systemic lupus erythematosus (JSLE), neuropsychiatric lupus (NPSLE), seizures

## Abstract

Juvenile Systemic Lupus Erythematosus (JSLE) is a rare and chronic multisystem autoimmune condition with an accelerated course compared with adult‐onset SLE. Its heterogeneity of presentation and overlap with infectious and inflammatory conditions frequently hinders diagnosis, especially in areas where tuberculosis is endemic. A 15‐year‐old male patient came to the Emergency Department with his sixth attack of generalized tonic–clonic seizures within 20 days. He had a history of 8 months with complaints of joint pain, intermittent fever, rash, alopecia, and lymphadenopathy of the cervical and inguinal region. Proteinuria and abnormal liver function tests were found in laboratory investigations. Neuroimaging was found to be hyperintense lesions in the right parieto‐temporal area. The autoimmune profile was found to have positive ANA, anti‐Sm, and anti‐RNP antibodies with decreased complement C4. Diagnosis of Juvenile Systemic Lupus Erythematosus (JSLE) along with lupus cerebritis was established. He was treated with corticosteroids, immunosuppressants, and antiepileptics. This case highlights the diagnostic difficulty of JSLE in children who come with atypical or isolated neuropsychiatric symptoms. A false interpretation of a positive IGRA resulted in a provisional diagnosis of tuberculosis, thus delaying proper treatment. Autoimmune causes such as JSLE should be considered by clinicians in children with unexplained neurological manifestations and persistent multisystemic symptoms, particularly in areas where TB burden is high.

## Key Clinical Message

Juvenile Systemic Lupus Erythematosus (JSLE) can manifest with isolated neuropsychiatric features like recurrent seizures, causing diagnostic dilemmas, particularly in an endemic area for tuberculosis. All physicians need to highly consider autoimmune causes in a child with chronic multisystemic manifestations and unknown neurological abnormalities to make an early diagnosis and treatment.

## 1 Introduction

Juvenile Systemic Lupus Erythematosus (JSLE) is a rare, chronic autoimmune condition that occurs in children below the age of 18 years. It is distinguished by a broad spectrum of clinical manifestations that may affect multiple organ systems and may be manifested at varying degrees of severity [1]. JSLE, in comparison with adult‐onset SLE, shows higher rates of disease activity, organ damage, and occurrence of specific manifestations [2].

JSLE may be difficult to diagnose because it has a heterogeneous presentation and there are no specific diagnostic criteria, which may rely on those of adult‐onset SLE [3]. Early and proper diagnosis is essential in the management of the disease and to preclude serious complications since JSLE is more aggressive than its adult counterpart [4]. Multisystemic involvement is characteristic of JSLE, and almost all of the body systems are susceptible. The most reported symptoms are constitutional (e.g., fever, fatigue, weight loss), mucocutaneous (e.g., malar rash, mouth sores, hair balding), musculoskeletal (e.g., arthritis, arthralgia), and even hematological (e.g., anemia, leukopenia, thrombocytopenia). Among more severe manifestations, neuropsychiatric lupus (NPSLE) poses a serious concern, and a considerable number of pediatric individuals with SLE develop it. NPSLE is very diverse, with an equal variety of neurological and psychiatric symptoms, such as seizures, headaches, cognitive dysfunction, mood disorders, and psychosis, which may have a dramatic effect on the patient's quality of life and functional status [1, 5].

The following case presentation provides detail on the clinical course of a 15‐year‐old male with multiple and complicated features of JSLE, which included the involvement of the nervous system.

## 2 Case Presentation

A 15‐year‐old boy was brought to the Emergency Department with active generalized tonic–clonic seizures. This was his sixth episode in the past 20 days, each lasting approximately 20 min. He had no prior history of seizures. According to his parents, he was previously healthy, had achieved all developmental milestones on time, and was performing well academically. His vaccination history was up to date.

The patient had been unwell for the preceding 8 months. His symptoms began with persistent joint pains and a non‐productive cough. Evaluation at that time revealed a left‐sided pleural effusion, and a positive interferon‐gamma release assay (IGRA) led to empirical initiation of anti‐tuberculous therapy (ATT). Despite completing a full course of ATT, his condition did not improve. Over the subsequent months, he developed additional symptoms suggestive of multisystemic involvement: intermittent fever, a malar rash, a generalized maculopapular rash on the face, trunk, and limbs, patchy alopecia with visible scarring, and cervical and inguinal lymphadenopathy. Laboratory investigations during this period revealed proteinuria and deranged liver function tests. On arrival at the Emergency Department, the patient was semiconscious and actively seizing.

### 2.1 Examination Findings

On arrival at the Emergency Department, the patient was semiconscious and actively seizing, with generalized tonic–clonic activity. He was anemic in appearance with significant pallor, and palpable discrete nontender cervical and inguinal lymph nodes of mixed size. His temperature was 100°F, heart rate 110 beats per minute, respiratory rate 24 breaths per minute, and oxygen saturation 95% on room air.

Neurological examination was marked by a Glasgow Coma Scale (GCS) of 8/15 (E2, V2, M4). Bilaterally upgoing plantar reflexes and dilated but equally reactive pupils were present. Meningeal irritation was not present. Cutaneous findings were associated with a characteristic malar rash, maculopapular rash on the limbs, and oral mucosal ulceration of the buccal mucosa. Patchy scarring alopecia of the scalp. Musculoskeletal examination revealed bilaterally swollen, warm, and tender knee joints in congruence with inflammatory arthritis.

### 2.2 Investigations

Investigations at presentation revealed mild anemia with hemoglobin level of 11.3 g/dL and leukocytosis with total leukocyte count 12 × 10^9^/L, while platelet count was normal at 157 × 10^9^/L. Serum electrolytes were normal, i.e., sodium 139 mmol/L and potassium 3.8 mmol/L. Blood glucose was 100 mg/dL. Liver function tests revealed marked hypertransaminasemia as SGPT was 168 U/L and hyperbilirubinemia due to albumin concentration of 4.04 mg/dL which indicated liver involvement. The renal function was normal with serum creatinine 0.6 mg/dL and urea 28 mg/dL. Coagulation profile was within normal limits with prothrombin time of 14.9 s and activated partial thromboplastin time of 28 s. Serum calcium was 8.5 mg/dL. Malaria parasite ICT, dengue antigen, and antibody functions were negative, and blood cultures remained sterile after 5 days' incubation.

Immunological assessment was characterized by low complement C4 that was 5 mg/dL (normal range 10–40 mg/dL) with normal C3 that was 93 mg/dL (normal range: 90–180 mg/dL), suggesting active immune complex disease. Procalcitonin was raised at 89.68 ng/mL (normal < 0.05 ng/mL), while anti‐cyclic citrullinated peptide (anti‐CCP) antibodies were negative. Urinalysis revealed proteinuria, adding weight to renal involvement.

Cerebrospinal fluid (CSF) analysis, performed after exclusion of elevated intracranial pressure, revealed lymphocytic predominance (75% lymphocytes), protein of 49 mg/dL, and glucose of 77 mg/dL. This was in the direction of aseptic inflammation as against bacterial infection.

Autoimmune profile also confirmed the diagnosis since it was positive for antinuclear antibody (ANA) with speckled pattern (++), positive for anti‐SS‐A (++), Ro‐52 (+), anti‐nRNP/Sm (++), and anti‐Sm (++). Anti‐neutrophil cytoplasmic antibody (ANCA), anti‐mitochondrial antibody (AMA), and anti‐smooth muscle antibody (ASMA) were negative.

### 2.3 Imaging Findings

**FIGURE 1:**
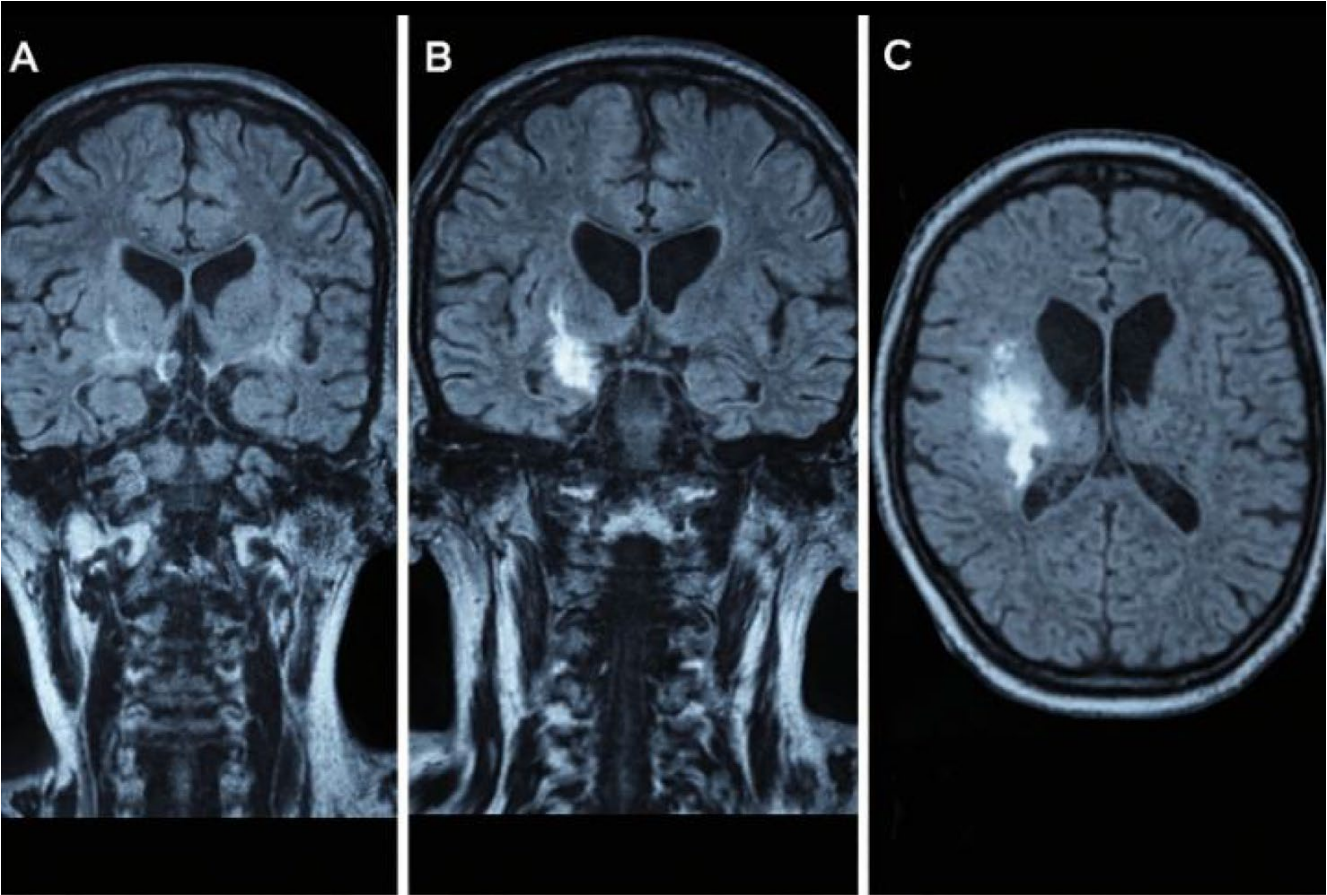
Brain MRI (T2/FLAIR sequences). (A) Coronal section demonstrating hyperintense signal in the deep white matter of the right parieto‐temporal region. (B) Adjacent coronal slice confirming the hyperintense lesion consistent with inflammatory involvement suggestive of lupus cerebritis. (C) Axial view demonstrating the corresponding white‐matter abnormality.

Magnetic resonance imaging (MRI) of the brain (T2/FLAIR sequence) showed abnormal hyperintense signals from right parieto‐temporal deep white matter, which is suggestive of an inflammatory or autoimmune process similar to lupus cerebritis (Figure 1).

### 2.4 Diagnostic Assessment

Based on the immune profile of the patient, imaging details, Cerebrospinal fluid (CSF) analysis, CBC report and clinical presentation along with systemic features a diagnosis of Juvenile Systemic Lupus Erythematosus (JSLE) with lupus cerebritis and multisystem involvement was made.

### 2.5 Management

The patient was admitted for multidisciplinary care. Intravenous methylprednisolone (30 mg/kg/day for 3 consecutive days) was administered, followed by oral prednisolone at 1 mg/kg/day in divided doses with a gradual taper planned. Hydroxychloroquine was initiated at 5 mg/kg/day to control cutaneous and joint manifestations and to reduce disease flares. Given the multisystemic involvement, immunosuppressive therapy with azathioprine was commenced at 2 mg/kg/day orally as a steroid‐sparing agent. Supportive therapy included calcium and vitamin D supplementation for bone health and dietary modification to address proteinuria. Regular monitoring of complete blood count, liver and renal function, and urinalysis was implemented.

### 2.6 Follow‐Up and Outcomes

Progress noted in neurological status during stay, after starting corticosteroids alongside antiseizure medication. Seizures stopped, awareness increased step by step. Skin lesions and joint pain started fading over time. At 4 weeks follow‐up, no further convulsions occurred; rash less intense, joints more functional. Tests revealed lower inflammation levels, internal systems holding steady. Observation moving forward arranged through specialists in immune‐related conditions and nervous system disorders. Monitoring intended for tracking changes in illness behavior and how well therapies work.

## 3 Discussion

Juvenile‐onset Systemic Lupus Erythematosus (JSLE), which develops before 16 years, constitutes about 10 to 20% of all SLE occurrences. Notable pathophysiological differences between juvenile‐onset and adult‐onset SLE are indicated by age‐related disparities in gender distribution, higher disease severity in children and adolescents, and early childhood monogenic forms [6]. This case report describes the challenging course of JSLE in a young man aged 15 years, illustrating all the difficulties of diagnosis and a described extreme involvement of the neurologic system, in this case, lupus cerebritis, as the first expressive symptom. The fact that the patient presented with repeated generalized tonic–clonic seizures and continued fever, rash, and joint pain with the initial misdiagnosis of tuberculosis reflects the heterogeneous nature of JSLE.

Occurring rarely, juvenile systemic lupus erythematosus affects about 0.3 to 0.9 out of every 100,000 kids each year, while around 3 to 9 per 100,000 live with it globally. More severe than forms seen in adults, this condition often brings stronger symptoms and greater chances of harm to organs—especially kidneys and nerves. Because waiting too long to identify it can cause lasting damage and worsen health outcomes, spotting signs early matters greatly. Even though better blood tests and scanning methods now help catch it sooner, its unpredictable nature still confuses doctors, especially where medical tools and expertise are scarce [6, 7].

In the case, a major obstacle to diagnosis was that the patient initially presented with severe neurological manifestations, especially seizures, which needed emergency treatment. Despite neuropsychiatric complication being an established complication of JSLE, it is quite unusual to see a case where it is the initial and most severe presenting symptom [1, 5]. The diagnostic process was also impaired by an inconclusive false‐positive interferon‐gamma release assay (IGRA). Such a finding may be highly indicative of a tuberculosis infection in areas such as Pakistan, where tuberculosis is endemic. This resulted in the commencement of empirical anti‐tuberculous treatment, which, unfortunately, resulted in the erroneous diagnosis of JSLE. The case illustrates a major clinical pitfall, which is not paying attention to the single positive test but thoroughly connecting the clinical picture and microbiological verification of the results to avoid misdiagnosis and delay the necessary treatment [4].

Upon the patient's presentation with seizures and systemic screening, a wide spectrum of the differential diagnosis was considered. As there was fever and convulsions, shortly infectious causes with specific attention to central nervous system diseases such as meningitis or encephalitis were high on the list. This is consistent with the diagnostic methodology that should be followed in the case of neuropsychiatric lupus, in which ruling out infections is the first and crucial step [8].

Lumbar puncture and blood cultures were carried out to rule out the infectious and inflammatory conditions of the CNS. The presence or absence of other autoimmune encephalitides‐specific antibodies ended up narrowing the diagnosis to JSLE. The fever, rash, and arthralgia, which persisted with anti‐tuberculous therapy, warranted further thorough workup of systemic inflammatory or autoimmune diseases. The typical neurological manifestations, or the seizures, necessitated the ruling out other likely causes of the condition in an adolescent, such as epilepsy or metabolic or structural brain anomalies. The neuropsychiatric manifestations of JSLE include one of the most common and serious complications, seizures, which require a detailed examination to exclude the mimics [7]. In this context, the findings of the MRI were critical. The fact that abnormal hyperintense signals in the deep white matter of the right parieto‐temporal region were discovered also led to the diagnosis indicating the presence of an inflammatory or autoimmune process in the brain. Although these MRI findings can be generally non‐specific, they are representative of a typical pattern seen in CNS lupus, representing regions of ischemia or inflammation initiated by underlying vasculopathy or antibody‐mediated neuronal damage in the disease [8]. The findings in themselves, when considered along with the changing clinical appearance of the patient and with the positive serology results, made a strong case in favor of a diagnosis of lupus cerebritis.

The markedly elevated liver enzymes observed in this patient may reflect hepatic involvement secondary to systemic inflammation or autoimmune activity associated with JSLE. Mild hepatic dysfunction has been described in lupus patients and may result from immune‐mediated hepatocellular injury or systemic inflammatory response. Similarly, the elevated procalcitonin level initially raised suspicion for severe bacterial infection. However, procalcitonin elevation has occasionally been reported in autoimmune inflammatory states, including severe lupus flares, and may not always indicate bacterial infection. In the present case, negative cultures and cerebrospinal fluid findings supported a non‐infectious inflammatory etiology [9, 10].

This case overlaps with the available literature that discusses the varying and sometimes debilitating neuropsychiatric manifestations of JSLE. Most clinical descriptions of NPSLE include more subtle neurologic manifestations like headache, cognitive impairment, or an affective disorder, with seizures being a late finding [11]. Nevertheless, the strength of the presenting problem of recurrent generalized tonic–clonic seizures as an early and incapacitating symptom of the condition, prior to the definitive diagnosis of JSLE, also makes this case of special interest, but more so given the absence of alternative neuropsychiatric manifestations and with the patient otherwise remaining in a normal psychological state.

A child's health can deteriorate sharply if JSLE goes unnoticed, opening the door to severe issues like kidney inflammation, lasting nerve system harm, heart‐related problems, or more frequent symptom surges. When brain or mental function is impacted and care stalls, fits may continue unchecked, thinking slows, and nervous system decline becomes harder to reverse. Spotting signs sooner allows treatment with immune‐calming drugs to begin quickly—this shift often lowers how active the illness remains, shields vital organs, and shapes a better future for young patients caught in its path [2, 6, 7].

## 4 Conclusion

This case highlights the importance of accurate and timely diagnosis in juvenile patients presenting with systemic and neurological symptoms. This patient was initially misdiagnosed with tuberculosis based solely on a positive interferon gamma release assay (IGRA), without microbiological or histopathological confirmation. Positive IGRA results are common in Pakistan and reflect prior exposure. This led to a significant delay in diagnosing juvenile systemic lupus erythematosus (JSLE). It is important for clinicians to maintain a high index of suspicion for autoimmune diseases in patients with unexplained and persistent symptoms. Funding Statement and Affiliations.

All authors certify that they have no affiliation with or involvement in any organization or entity with any financial interest or non‐financial interest in the subject matter or materials discussed in this manuscript.

## Author Contributions

**Shafaq Nazia:** conceptualization, data curation, supervision. **Aliza Andaleeb:** data curation, formal analysis, investigation, software, visualization, writing – original draft. **Abdul Moiz:** methodology, project administration, validation, visualization, writing – original draft. **Hareem Ahmed:** data curation, formal analysis, investigation, project administration, resources, validation, visualization, writing – original draft. **Muhammad Hassaan Javaid:** writing – review and editing. **Abubakr Mahmoud:** formal analysis, investigation, methodology, project administration, supervision, validation, visualization, writing – review and editing.

## Funding

The authors have nothing to report.

## Consent

Written informed consent was taken from the patient's guardian.

## Conflicts of Interest

The authors declare no conflicts of interest.

## Data Availability

The authors have nothing to report.
